# Biotransformation of Isoflavone Using Enzymatic Reactions

**DOI:** 10.3390/molecules18033028

**Published:** 2013-03-06

**Authors:** Changhyun Roh

**Affiliations:** Division of Biotechnology, Advanced Radiation Technology Institute (ARTI), Korea Atomic Energy Research Institute (KAERI), 1266, Sinjeong-dong, Jeongeup, Jeonbuk 580-185, Korea; E-Mail: chroh@kaeri.re.kr; Tel.: +82-63-570-3133; Fax: +82-63-570-3139

**Keywords:** enzyme, isoflavone, bioactive compound, hydroxylation, biocatalyst

## Abstract

The roles of cytochrome P450 monooxygenases (CYPs) from *Streptomyces* spp. which are called the “treasure islands” for natural products for medicine and antibiotics are not well understood. Substrate specificity studies on CYPs may give a solution for elucidation of their roles. Based on homology sequence information, the CYP105D7 of a soluble cytochrome P450 known as heme protein from *Streptomyces avermitilis* MA4680 was expressed using the T7 promoter of the bacterial expression vector pET24ma, over-expressed in *Escherichia coli* system and characterized. An engineered whole cell system for daidzein hydroxylation was constructed using an exogenous electron transport system from ferredoxin reductase (PdR) and ferredoxin (Pdx). Also, an *in vitro* reaction study showed the purified CYP105D7 enzyme, using NADH-dependent-reducing equivalents of a redox partner from *Pseudomonas putida*, hydroxylated daidzein at the 3' position of the B ring to produce 7,3,'4' trihydroxyisoflavone. The hydroxylated position was confirmed by GC-MS analysis. The turnover number of the enzyme was 0.69 μmol 7,3,'4'-trihydroxyisoflavone produced per μmol P450 per min. This enzyme CYP105D7 represents a novel type of 3'-hydroxylase for daidzein hydroxylation. A P450 inhibitor such as coumarin significantly (ca.98%) inhibited the daidzein hydroxylation activity.

## 1. Introduction

Recently, *ortho*-dihydroxyisoflavones (7,8,4'-trihydroxyisoflavone, 6,7,4'-trihdyroxyisoflavone and 7,3',4'-trihydroxyisoflavone) are of growing scientific interest to enhance their health-related applications in humans. The isoflavone compounds hydroxylated at a specific *ortho* position have potent antioxidant properties that contribute to their cholesterol-lowering effect, cardiovascular protective, anti-tumor and anti-carcinogenic properties [1,2,3,4,5,6,7]. Furthermore, these compounds are known to display anti-inflammatory and anti-allergenic activity, and acts as potent tyrosinase inhibitors which is associated with inhibition of melanin formation [6,8]. According to many biological effect studies, 7,3',4'-trihydroxyisoflavones have received much attention in recent years due to their potentially beneficial estrogenic, antioxidant, and anticancer properties in humans [1,9]. Furthermore, 7,3',4'-trihydroxyisoflavone exhibited more effective antioxidant activity using the oxygen radical absorbance capacity (ORAC) assay as well as the oxidation of low-density lipoproteins (LDL) assay [6,10].

In general, monooxygenases act as oxidants in a range of reactions, including the transfer of molecular oxygen to X-H bonds of a substrate. Among the monooxygenase enzymes, P450s are proficient at highly regio-specific hydroxylation reactions. Cytochromes P450 (CYPs) are heme- containing enzymes that play a major role in the metabolism of xenobiotics, including drugs, carcinogens, and environmental chemicals, as well as endogenous compounds such as steroids and fatty acids. This enzyme is very common in eukaryotic and prokaryotic systems and responsible for various metabolizing activities as a monooxygenase. The usefulness of the P450 monooxygenases consists in the importance of their bioconversion activities of water insoluble chemicals into soluble ones by the introduction of single oxygens via hydroxylation reaction [11,12,13,14]. Most P450s catalyze two-electron oxidation reactions of organic substrates coupled to O-transfer [Equation (1)] Dioxygen and the substrate (RH) bind at the heme active site and electrons from NADPH act to cleave the O–O bond leaving a high valent oxo-heme moiety that is capable of O-insertion into an otherwise inert C–H bond:

NAD(P)H + RH + O_2_ + H^+^ → NAD(P)^+^ + ROH + H_2_O (1)

Nowadays, many of the P450 monooxygenases have been reported in prokaryotes like the Actinomycetes, which have been intensively studied for their importance as industrial microorganisms in producing various kinds of antibiotics and other bioactive substances. By an appropriate involvement of P450 enzymes, many bioactive compounds are produced in Actinomycetes. Taylor *et al*. reported that CYP105D1 from *Streptomyces griseus* has catalytic capacity in the hydroxylation of the steroid testosterone, warfarin and naphthoflavone compounds [15].

Recently, due to the completion of the genome sequences of many microorganisms, it appears to be eminently possible to capture specific genes from the whole genome of a particular microorganism. Especially, Actinomycetes strains produce many useful natural compounds and metabolites as well as polyketide biosynthesis [16,17,18]. Among these strains, *Streptomyces avermitilis* MA4680 produces many useful antibiotics such as avermectin, oligomycin, geosmin, filipin, and pentalenolactone. The genome sequence of this strain was published by the Omura group in 2003 [19], revealing 33 putative CYP genes. Consequently, based on sequence homology information from *Streptomyces avermitilis* MA4680 genome, it was possible to elucidate the characterization of CYP105D7, which is involved in daidzein hydroxylation. The goal of this study was to examine the capability of engineered metabolism to transform daidzein for the purposes of converting it into 7,3',4'-trihydroxyisoflavone.

## 2. Results and Discussion

### 2.1. Chemical Structure of 7,3',4'-Trihydroxyisoflavone from Daidzein

**Scheme 1:**
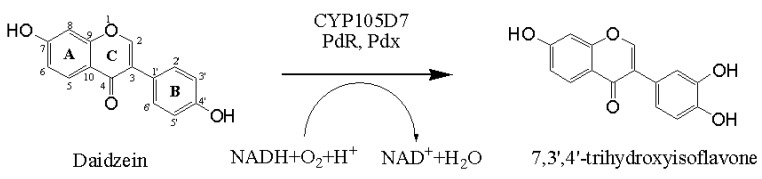
Representative scheme of the hydroxylation in the 3'-position of the daidzein catalyzed by CYP105D7 with a suitable redox partner.

The chemical structure of daidzein, known as a phytoestrogen, is very similar that of the human hormone estrogen. The basic structural unit of the daidzein comprises two benzene rings (A and B), which are linked via a heterocyclic pyrone ring (C). Thus, the structure of 7,3',4'-trihydroxyisoflavone implies the hydroxylation of daidzein at position C3' of the B-ring. The strategy for daidzein hydroxylation is represented in Scheme 1.

### 2.2. Identification and Functional Construction of CYP105D7 and Redox Partner in E. coli System

**Figure 1:**
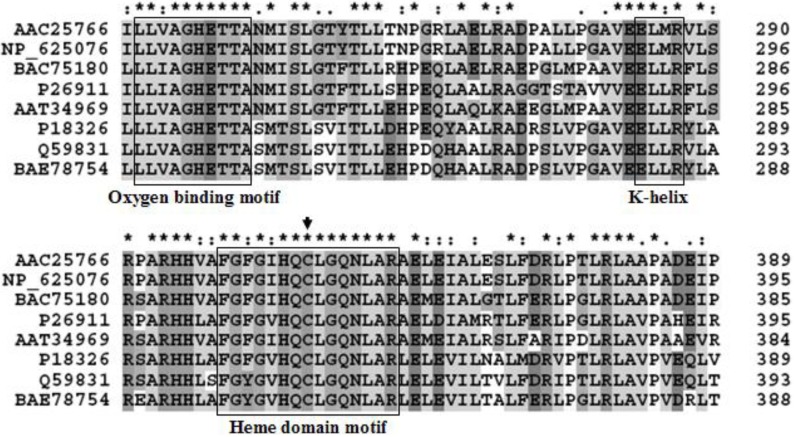
Cytochrome P450 alignments of the amino acid sequence with other bacterial strains. P26911; CYP from *Streptomyces griseus*, BAC75180; CYP from *Streptomyces avermitilis* MA4680, AAC25766; CYP from *Streptomyces lividans*, NP_625076; CYP from *Streptomyces coelicolor* A3(2), AAT34969; CYP from *Streptomyces tubercidicus*, P18326; CYP from *Streptomyces griseolus*, BAE78754; CYP from *Streptomyces sp*. TM-7, Q59831; CYP from *Streptomyces carbophilus*. Multiple sequence alignment of conserved motifs in P450 enzymes identified. Both oxygen-binding motif and heme domain sequences as well as the EXXR motif in the K helix are shown. Amino acids conserved in the P450s listed are shown. The Cys residue that coordinates the heme is indicated by an arrow. Alignments were performed with the CLUSTAL X 1.83. (*) Identical residues in the different enzymes; (:) conserved substitutions; (.) semi-conserved substitutions.

**Figure 2:**
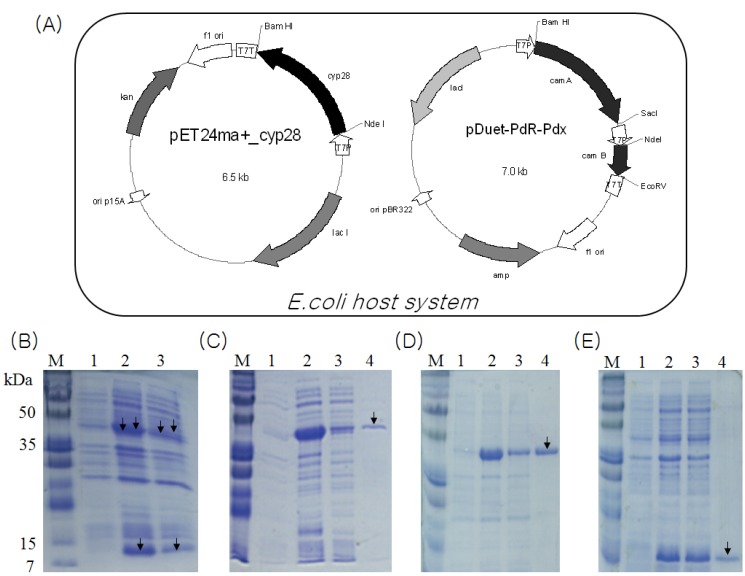
(**A**) A schematic CYP105D7 and PdR/Pdx coexpression plasmids in *E. coli* system. CYP105D7 is encoded on the 6.5 kb constructed pET24ma+_cyp28 under control of a T7 promoter with IPTG induction system. The 7.0 kb plasmid pDuet-PdR-Pdx contains two copies of the T7 promoter and a transcription terminator; (**B**) SDS-PAGE 13% gel showing the coexpressed proteins of CYP105D7 and PdR/Pdx. lane M, protein marker; lane 1, before induction form of CYP105D7 and PdR/Pdx; lane 2, total form of CYP105D7 and PdR/Pdx; lane 3, soluble form of CYP105D7 and PdR/Pdx; (**C**) SDS-PAGE 12% gel showing CYP105D7 with His-tag. M, protein marker; lane 1, before induction form of CYP105D7; lane 2, total form of CYP105D7; lane 3, soluble form of CYP105D7; lane 4, His-Tag form of CYP105D7; (**D**) SDS-PAGE 12% gel showing PdR with His-tag. M, protein marker; lane 1, before induction form of PdR; lane 2, total form of PdR; lane 3, soluble form of PdR; lane 4, His-Tag form of PdR; (**E**) SDS-PAGE 13% gel showing Pdx with His-tag. M, protein marker; lane 1, before induction form of Pdx; lane 2, total form of Pdx; lane 3, soluble form of Pdx; lane 4, His-Tag form of Pdx. Arrow represents protein, respectively.

Since CYP105D1 used naphthoflavone as a substrate, its sequence homology was searched on a NCBI database. Eight high homology P450 genes were found in the database, such as P450 from *Streptomyces griseus* (GenBank accession no. P26911), P450 from *Streptomyces avermitilis* MA4680 (GenBank accession no. BAC75180), P450 from *Streptomyces lividans* (GenBank accession no. AAC25766), P450 from *Streptomyces coelicolor* A3(2) (GenBank accession no. NP_625076), P450 from *Streptomyces tubercidicus* (GenBank accession no. AAT34969), P450 from *Streptomyces griseolus* (GenBank accession no. P18326), P450 from *Streptomyces* sp. TM-7 (GenBank accession no. BAE78754) and P450 from *Streptomyces carbophilus* (GenBank accession no. Q59831). Among them, one (GenBank accession no. BAC75180) was chosen because it was translated well in the *E. coli* host system. The size of the gene orf (*cyp28*) was 1,215 bp, encoding a cytochrome P450 protein (CYP105D7) with molecular mass of 44.5 kDa. The sequence analysis by Clustal X is shown in Figure 1. Both the oxygen-binding motif and heme domain sequences as well as the EXXR motif in the K helix are shown with a box. The three component electron transfer combination was constructed in the *Escherichia coli* system for whole cell-mediated daidzein hydroxylation by coexpression of the corresponding coding sequences from two plasmids (Figure 2A). The developed system in this study was inserted in two plasmids containing different selection markers to enable the construct to be coexpressed stably in an *E. coli* strain. The presence of both plasmids in the expression strain was assured by growing the LB media containing the antibiotics kanamycin and ampicillin. In this coexpression system, all three proteins involved in the *E. coli* system as a host were organized.

### 2.3. Overexpression and Purification of CYP105D7, PdR and Pdx

Heterologous coexpression of CYP105D7, PdR and Pdx in the *E. coli* system was performed. Three proteins with molecular weight of about 45 kDa (P450), 46 kDa (ferredoxin reductase) and 12 kDa (ferredoxin) were visible on Coomassie staining of SDS-PAGE (Figure 2B). The CYP105D7 protein was purified by a single chromatography step on a Ni^2+^ chelate affinity chromatography. N-terminal His-tag P450bioI showed a molecular mass of approximately 45 kDa in a SDS-PAGE (Figure 2C). The amount of purified protein was calculated to be approximately 200 μM·L^−1^ using the extinction coefficient for oxidized CYP105D7 of ε418 = 181 mM^−1^ cm^−1^. Also, purification of the N-terminal His-tagged ferredoxin reductase and ferredoxin was performed using the same procedures as described above (Figure 2D,E).

### 2.4. Characterization of Recombinant CYP105D7 and Kinetic Parameter

**Figure 3:**
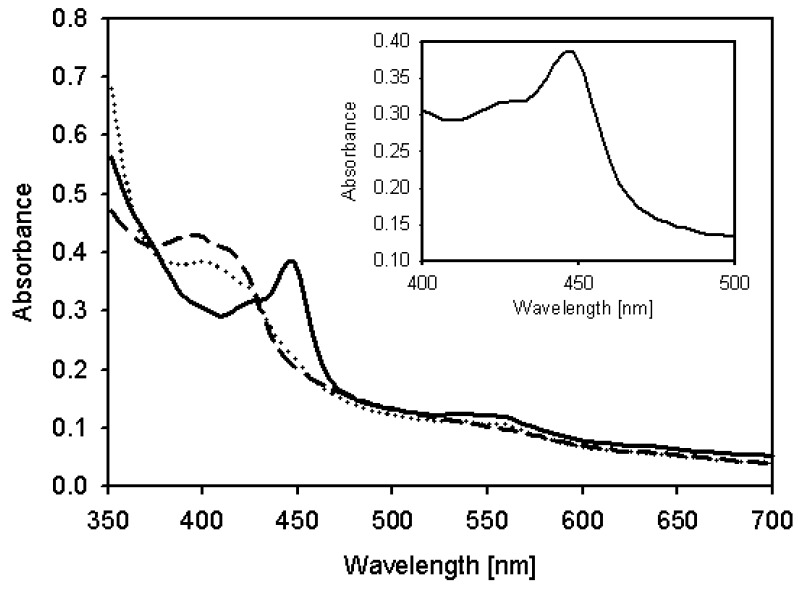
UV-visible absorbance spectra of expressed CYP105D7. Dashed line: oxidized CYP105D7, Dotted line: Reduced form with dithionite, Solid line: CO-bound form spectra with dithionite. The insert represents the CO difference spectra.

Their activities of the coexpressed proteins were examined by UV-Vis spectroscopy, which revealed the typical characteristics of P450, a heme Soret peak at 420 nm (low spin) in the absence of carbon monoxide. The spectrum of the dithionite-reduced and CO-bound form showed the well known absorption maximum at 450 nm (Figure 3). In Table 1, we showed the apparent specificity constants (kcat/Km) by fitting the concentration-dependent data to the linear region of the Michaelis-Menten Lineweaver-Burke curve. The kcat/Km value for daidzein hydroxylation is approximately 0.69 (Km = 21.83 μM, kcat = 15.01 min^−1^). For kinetic parameters, CYP105D7 with a redox partner showed a typical hyperbolic profile which means the dependency of reaction rate on substrate concentration, corresponding to classic Michaelis-Menten equation. In this system, NADH was used as general electron donor in the *in vitro* reaction.

**Table 1:** Catalytic kinetic analysis for NADH-dependent hydroxylation of daidzein from CYP105D7.

Substrate	*K_m_* [μM]	*kcat* [min^−1^]	*kcat*/*K_m_* [μM^−1^/min^−1^] *
Daidzein	21.83 ± 6.3	15.01 ± 0.6	0.69

* Reaction was performed in potassium phosphate buffer at pH 7.5 in the presence of 10 mM NADH; The same protein concentration of PdR and Pdx was added for reaction.

### 2.5. Hydroxylation of Daidzein by CYP105D7 *in Vivo* and *in Vitro* System

**Figure 4:**
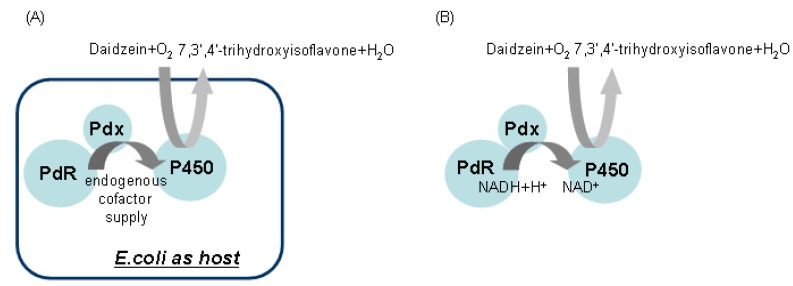
Schematic representation of the *in vivo* and *in vitro* reaction designed for daidzein hydroxylation.

**Figure 5:**
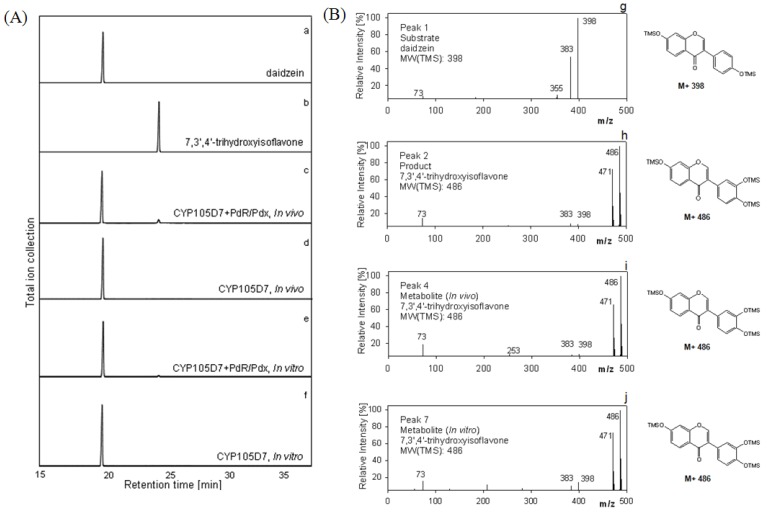
Gas Chromatography-Mass Spectroscopy analyses for daidzein hydroxylation *in vivo* and *in vitro* reaction. (**A**) GC chromatograms of the hydroxylated metabolite of daidzein. a, authentic daidzein; b, authentic 7,3',4'-trihydroxyisoflavone c, *in vivo* reaction with heterologously expressed P450 and redox partner; d, *in vivo* reaction with P450; e, *in vitro* reaction with P450 and PdR/Pdx; f, *in vitro* reaction with P450; (**B**) Representative electron impact mass spectra of TMS derivatives of daidzein and metabolite. g, mass spectrum of authentic daidzein (Peak, 1); h, mass spectrum of authentic 7,3',4'-trihydroxyisoflavone, (Peak, 2); i, mass spectrum of reactant, 7,3',4'-trihydroxyisoflavone (Peak, 4); j, mass spectrum of reactant, 7,3',4'-trihydroxyisoflavone (Peak, 7). Peak 3, 5, 6 and 8 represent mass spectra of daidzein. Retention times: daidzein, 19.5 min and 7,3',4'-trihydroxyisoflavone, 24.1 min.

For recombinant whole cell reactions, the schematic strategy for daidzein hydroxylation was orchestrated as shown in Figure 4A. For the *in vitro* system, the delivery of the two electrons from NADH to the P450 must be organized as seen in Figure 4B as well. Daidzein was hydroxylated to 7,3',4'-trihydroxyisoflavone, indicating PdR and Pdx are functional as active artificial electron transport partners for CYP105D7 activity. The identification of the hydroxylated products detected in the whole cell and enzyme reaction was performed. The product, 7,3',4'-trihydroxyisoflavone, was clearly detected by HPLC analysis in the *in vivo* and *in vitro* reactions (data not shown). In the *in vitro* system, there was no activity when the NADH cofactor was omitted (data not shown). To further verify the hydroxylation position of the product formed from daidzein, GC/MS analysis was performed after trimethylsilylation with BSTFA. The reaction product showed identical retention time (RT = 24.1 min) to that of authentic 7,3',4'-trihydroxyisoflavone (Figure 5A). In the mass spectrum of the product an *m/z* 486 peak for 7,3',4'-trihydroxyisoflavone was observed, indicating the hydroxylation at C3' of B-ring position (Figure 5B). The endogenous redox partner from *E. coli* did not function and give no activity in daidzein hydroxylation in the whole cell reaction with CYP105D7. Figure 5 shows GC-MS mass spectra of daidzein and the 3'-hydroxylated daidzein product obtained with PdR and Pdx.

### 2.6. Effect of P450 Inhibitors on the Hydroxylation of Daidzein

The effect of common P450 inhibitors such as coumarin, erythromycin and ketoconazole [20,21,22] on the hydroxylation of daidzein was examined by adding 0.5 mM of these inhibitors. The results are shown in Table 2. Among all the P450 inhibitors examined the hydroxylation activity was significantly inhibited by coumarin. Ketoconazole inhibited the reaction by 86%. Erythromycin inhibited the daidzein hydroxylation reaction by 37%.

**Table 2:** Effect of P450 inhibitors on hydroxylation of daidzein.

P450 inhibitor *	Reactive activity (%)
None	100
Coumarin	2
Erythromycin	63
Ketoconazole	14

* Each inhibitor reaction was performed at the final concentration of 50 μM; The reaction of daidzein was performed at 30 °C.

## 3. Experimental

### 3.1. Chemicals

Daidzein, 7,3',4'-trihydroxyisoflavone, quinidine, coumarin and erythromycin were obtained from Sigma-Aldrich Chemical Co (Yongin, Korea). Ketoconazole and *N*,*O*-bis(trimethylsily)trifluoroacetamide were purchased from Fluka (Yongin, Korea). All other chemicals were of the highest grade available.

### 3.2. Plasmid Construction

For the co-expression system; the pET24ma vector was used for construction of the P450-plasmid pET24ma_cyp28. The other expression vector (Cat. no. 71146-3; Novagen, Madison, WI, USA) was constructed for the redox partners ferredoxin reductase (PdR) and ferredoxin (Pdx)-plasmid pDuet-PdR-Pdx. For his-tag purification; pET28a+ vector (Cat. no. 69864-3; Novagen) was used. The plasmid construction was carried out with *E. coli* (DH5α, Novagen). The expression of target genes were performed using *E. coli* BL21 (DE3, Novagen).

### 3.3. Cloning of CYP105D7 from Streptomyces avermitilis MA4680

The strain, *Streptomyces avermitilis* MA4680, was obtained from the Microbial Resources Center (SNU, Seoul, South Korea). The genomic DNA of strain was prepared by G-spin genomic DNA extraction kit (Intron, Seoul, Korea) and used as template for PCR. The P450 gene was amplified by PCR using a set of specific primers; sense, 5'-CTG*CATATG*ACAGAGCCCGGTACGT-3'; antisense, 5'-CAT*GGATCC*TTACCAGGTCACGGGGAGTTC-3', where italic letters indicate restriction enzyme site NdeI and BamHI, respectively. The PCR reaction conditions as follows: 35 cycles of denaturation for 1 min at 94 °C, annealing for 1 min at 68 °C, and extension for 1 min 30 s at 72 °C. The PCR product was cloned into NdeI/BamHI sites of expression vector pET24ma. For His-tag plasmid of P450, it was N-terminally attached (Novagen). Also, it was attached *N*-terminally at expression vector pET28a+ for cyp28.

### 3.4. Coexpression of CYP105D7 and Redox Partner for *in Vivo* System

The plasmids of P450 and redox partner were transformed together into *E.coli* BL21(*DE3*). The transformant was grown in Luria-Bertani (LB) medium containing 100 μg·mL^−1^ of kanamycin and ampicillin at 37 °C until the cell concentration reached to an OD_600nm_ of 0.6, and isopropyl-thio-β-D-galactopyranoside (IPTG) and δ-aminolevlunic acid as heme precursor were added to a final concentration of 0.5 mM, followed by growing for overnight at 28 °C. Cells were harvested by centrifugation and used for direct reaction.

### 3.5. Purification of CYP105D7 and Redox Partner for *in Vitro* System

For His-tag purification of P450, ferredoxin reductase and ferredoxin, each cells were washed three times using Phosphate Buffered Saline (PBS) containing 137 mM NaCl, 2.7 mM, 10 mM Na_2_HPO_4_ and 2 mM KH_2_PO_4_. Then, cells were harvested by centrifugation at 4,000 rpm for 30 min at 4 °C and resuspended in sonication buffer (pH 7.5) containing 100 mM Na_2_HPO_4_, 2 mM EDTA and 0.5 mM PMSF, 1 mM DTT, 1 mM MgCl_2_. Cells were disrupted by Sonicator (Misoni S3000, Canton, MA, USA). The cell debris was removed by centrifugation at 15,000 rpm for 30 min. The clear supernatant was collected and the soluble proteins (P450, PdR and Pdx) were purified with his-tag affinity column (QIAGEN GmbH, Hilden, Germany). Their proteins with (His)_6_-tag were N-terminally attached using a Ni-NTA resin (QIAGEN GmbH). The column-bound enzyme was washed with 50 mM potassium phosphate buffer (pH 7.5) containing 500 mM sodium chloride and 50 mM imidazole. Elution was performed by increasing the imidazole concentration to 250 mM. Imidazole and sodium chloride were removed by dialysis against 50 mM phosphate buffer (pH 7.5) overnight. The purity of the enzyme was estimated by SDS-PAGE in the eluted fractions. The protein concentration was determined as described by Bradford [23] with bovine serum albumin as the standard. Enzyme samples were supplemented with 50% glycerol and stored at −20 °C until use.

### 3.6. CYP105D7 Concentration Measurement and Absorption Spectra

The carbon monoxide (CO) difference spectra were measured on an UV/vis spectrometer (Spectronic, Genesys, Milton Roy, Rochester, NY, USA). The concentration of CYP105D7 was measured based on CO difference spectra, using an extinction coefficient of 91 mM^−1^ cm^−1^ at 450 nm [24]. The concentrations of redox partners were determined using extinction coefficients of 11.1 mM^−1^ cm^−1^ at 415 nm for ferredoxin and 10.0 mM^−1^ cm^−1^ at 454 nm for ferredoxin reductase [25].

### 3.7. Kinetic Study for Substrate Activity with CYP105D7

For kinetic study, NADH oxidation was measured by the absorption decrease at 340 nm with an UV/vis spectrometry (Thermo Labsystems, Beverly, MA, USA). A solution containing 100 µL of potassium phosphate buffer (pH 7.5), 20 μL of each substrate concentration solution in DMSO and 20 µL of enzyme solutions (CYP105D7, PdR and Pdx, 10 µmol/mL, respectively) was incubated for 5 min in 96 well microplate. The reaction was started by adding 20 μL of 10 mM NADH solution. For determination of kinetic parameters, a substrate concentration range of 31.25–500 μM was used. All data were fitted to the Lineweaver-Burke equation by linear regression. As a reference sample, NADH consumption rates at 340 nm were measured without substrate.

### 3.8. Daidzein Hydroxylation *in Vivo* and *in Vitro* System

For whole cell reaction of *E.coli* with coexpressed CYP105D7 and redox partner (ferredoxin reductase, ferredoxin), 50 mL of *E.coli* cell culture containing the overexpressed three enzymes was harvested and resuspended in 10 mL of LB broth containing 100 μg·mL^−1^ of kanamycin and ampicillin. Daidzein as substrate, at the final concentration of 50 μM was added into the resuspension cell, and was incubated on a shaking conical flask for about 6 h at 30 °C. For control, the overexpressed CYP105D7 without redox partner was performed at same conditions. The three volumes of ethyl acetate (Junsei, Cuisine, Japan) were then added into the reactants and vortexed thoroughly. The mixture was separated into aqueous and ethyl acetate phases by centrifugation, and the ethyl acetate phase was evaporated in centrifugal vacuum concentrator (BioTron, Bucheon, Korea). The dried reactant was dissolved in methanol (Merck, Darmstadt, Germany). For *in vitro* reaction using purified CYP105D7, ferredoxin reductase (PdR) and ferredoxin (Pdx), one hundred μg of the purified proteins were reacted with 0.5 mM NADH and 50 μM daidzein of final concentration in 100 mM potassium phosphate buffer (pH 7.2) at 20 °C for 2 h. For control, NADH was excluded from the reaction. 

### 3.9. High Performance Liquid Chromatography (HPLC) Analysis of Product

The reactant was analyzed using a HPLC (Autochro-3000, Young Lin, Anyang, Korea) equipped with a UV/Vis detector and a Waters symmetry C18 column (4.6 × 150 mm, 5.0 μM particle size, Waters, Milford, MA, USA). The flow rate was 1 mL·min^−1^ and the injection volume was 10 μL. The solvent used was acetonitrile (CH_3_CN):water:trifluoroacetic Acid (TFA) = 3:7:0.1% (v/v/v).

### 3.10. Gas Chromatography (GC)/Mass Spectrometry (MS) Analysis

For GC/MS analysis, reaction metabolites were converted to their TMS (trimethylsilyl) derivatives by heating for 20 min at 60 °C with BSTFA (*N*,*O*-bis(trimethylsily)trifluoroacetamide). GC/MS was carried out on a Finnigan MAT system (Gas chromatograph model GCQ, HP 19091J-433, Thermo Finnigan, Austin, TX, USA) connected to an ion trap mass detector. The TMS-derivatives were analyzed using a nonpolar capillary column (5% phenyl methyl siloxane capillary 30 m × 250 μm i.d., 0.25 μm film thickness, HP-5) and a linear temperature gradient (60 °C 1 min, 30 °C/min to 250 °C, hold for 10 min, 1 °C/min to 275 °C, and hold for 3 min). The injector port temperature was 100 °C. Scan spectrum was 100~600 *m/z* and mass spectra were obtained by electron impact ionization at 70 eV. The selected ion mode (SIM) was used for the detection of daidzein and its hydroxylated metabolite.

### 3.11. Effects of P450 Inhibitors on Hydroxylation of Daidzein

The used P450 inhibitors were coumarin, erythromycin and ketoconazole. The inhibition of daidzein hydroxylation by typical cytochrome P450 inhibitors was studied in the presence of 0.5 mM cytochrome P450 inhibitors with coexpressed recombinant enzymes. Inhibitors and daidzein were added at the same time for reaction, the conversion of product was determined by HPLC. As control, the same reaction condition without the addition of inhibitors was used. The extent of inhibition was analyzed by comparing quantative analysis of HPLC peak of product from control with those from reaction containing inhibitors.

## 4. Conclusions

In this study, our goal was to examine the capability of an engineered organism to transform daidzein for the purpose of converting it into 7,3',4'-trihydroxyisoflavone. We describe the construction of a whole cell bioconversion system for daidzein hydroxylation using a co-expressed redox partner system of ferredoxin reductase (PdR) and ferredoxin (Pdx) with CYP105D7. Here, we were able to identify a novel daidzein 3'-hydroxylase based on homology sequence information from substrate specificity studies. To the best of our knowledge, CYP105D7 is first microbial P450 for daidzein hydroxylation at the C-3' position of the B-ring. For hydroxylation, the system was composed of three components: NADH-dependent and FAD-containing ferredoxin reductase (FdR), ferredoxin (Fdx) as an electron-transfer protein and cytochrome P450, which acts as a monooxygenase in the microbial system. Here, we describe the design of a whole cell bioconversion system for daidzein using two plasmids composed of the electron transfer system PdR and Pdx and microbial CYP105D7. In a two plasmid approach, the genes of PdR and Pdx are constructed on the first plasmid, whereas the second plasmid contains the coding sequence for the expression of microbial CYP105D7. The ferredoxin protein delivers, consecutively, two electrons to the heme active site of the P450 in a carefully orchestrated process which ultimately leads to the observed hydroxylation activity. Redox equivalents are transferred from NADH via the proteins PdR and Pdx to enzyme CYP105D7 converting the daidzein hydroxylation. The CYP105D7 added hydroxyl group to B ring of daidzein to form 7,3,'4,-trihydroxyisoflavone in *E. coli* system with a redox partner. 

Through an *in vitro* reaction, we studied an isoflavone hydroxylase activity of CYP105D7 using an artificial electron transport system, consisting of ferredoxin reductase and ferredoxin. The chemical structure of the product was elucidated using GC-MS to confirm the location of the new hydroxyl group. We demonstrated the enzyme-tailored hydroxylation of daidzein, as a proposed method for structural modification of this substrate for further functional applications as a phytoestrogen compound. 
